# The structure of a reduced form of OxyR from *Neisseria meningitidis*

**DOI:** 10.1186/1472-6807-10-10

**Published:** 2010-05-17

**Authors:** Sarah Sainsbury, Jingshan Ren, Joanne E Nettleship, Nigel J Saunders, David I Stuart, Raymond J Owens

**Affiliations:** 1 The Oxford Protein Production Facility and Division of Structural Biology, Henry Wellcome Building for Genomic Medicine, University of Oxford, Roosevelt Drive, Oxford, OX3 7BN, UK; 2 The Bacterial Pathogenesis and Functional Genomics Group, The Sir William Dunn School of Pathology, University of Oxford, South Parks Road, Oxford, OX1 3RE. UK

## Abstract

**Background:**

Survival of the human pathogen, *Neisseria meningitidis*, requires an effective response to oxidative stress resulting from the release of hydrogen peroxide by cells of the human immune system. In *N. meningitidis*, expression of catalase, which is responsible for detoxifying hydrogen peroxide, is controlled by OxyR, a redox responsive LysR-type regulator. OxyR responds directly to intracellular hydrogen peroxide through the reversible formation of a disulphide bond between C199 and C208 in the regulatory domain of the protein.

**Results:**

We report the first crystal structure of the regulatory domain of an OxyR protein (NMB0173 from *N. meningitidis*) in the reduced state i.e. with cysteines at positions 199 and 208. The protein was crystallized under reducing conditions and the structure determined to a resolution of 2.4 Å. The overall fold of the *Neisseria *OxyR shows a high degree of similarity to the structure of a C199S mutant OxyR from *E. coli*, which cannot form the redox sensitive disulphide. In the neisserial structure, C199 is located at the start of helix α3, separated by 18 Å from C208, which is positioned between helices α3 and α4. In common with other LysR-type regulators, full length OxyR proteins are known to assemble into tetramers. Modelling of the full length neisserial OxyR as a tetramer indicated that C199 and C208 are located close to the dimer-dimer interface in the assembled tetramer. The formation of the C199-C208 disulphide may thus affect the quaternary structure of the protein.

**Conclusion:**

Given the high level of structural similarity between OxyR from *N. meningitidis *and *E. coli*, we conclude that the redox response mechanism is likely to be similar in both species, involving the reversible formation of a disulphide between C199-C208. Modelling suggests that disulphide formation would directly affect the interface between regulatory domains in an OxyR tetramer which in turn may lead to an alteration in the spacing/orientation of the DNA-binding domains and hence the interaction of OxyR with its DNA binding sites.

## Background

The *Neisseria *genus, of Gram-negative Betaproteobacteria, contains two closely related human pathogens, *N. meningitidis*, a leading cause of bacterial meningitis and septicaemia and *N. gonorrhoeae*, that causes the sexually transmitted infection, gonorrhoea [1]. Survival of bacteria like *Neisseria *depends upon an effective response to oxidative challenges from the host environment, for example, reactive oxygen species (ROS), superoxide (·O^2-^), hydrogen peroxide (H_2_O_2_) produced by phagocytes. To protect cellular components, including proteins and DNA, bacteria produce a range of enzymes, including catalases, peroxidases, oxidoreductases, and superoxide dismutases, whose expression is frequently controlled by redox-sensing transcription factors.

OxyR, widely known as a mediator of hydrogen peroxide-induced gene expression, is a redox-sensitive LysR-type transcription factor. Increased cellular hydrogen peroxide levels result in the oxidation of a pair of conserved cysteine residues present in the regulatory domain of OxyR [2] which leads in turn to the activation of transcription. In *E. coli*, OxyR regulates in excess of ten genes, including the hydrogen peroxide detoxifying enzyme catalase (*katG*), glutathione reductase (*gorA*), glutaredoxin 1 (*grxA*) and thioredoxin 2 (*trxC*) [3,4]. The neisserial OxyR which shares 37% amino acid sequence identity with its *E. coli *orthologue appears to control expression of a smaller regulon comprising the genes encoding catalase (*kat*), glutathione oxidoreductase (*gor *) and peroxiredoxin oxidoreductase (*prx*) [5]. The OxyR regulon appears to have an important role in pathogenesis since *N. gonorrhoeae *strains lacking either *oxyR, prx *or *gor *are deficient in both biofilm formation and show reduced survival in cervical epithelial models [5]. In fact, there are now several reports of OxyR being important for biofilm formation in other bacteria including *Pseudomonas aeruginosa *and *Tannerella forsythia *[6,7].

Crystal structures of the regulatory domains of wild type and a C199S mutant of *E. coli *OxyR have been reported. These structures, corresponding to oxidised and 'pseudo' reduced forms respectively, indicated that oxidation of OxyR leads to a local rearrangement of the C-terminal regulatory domain of the protein and formation of an intramolecular disulphide bridge between C199 and C208 [2]. Kim *et al*. [8] raised the possibility that the thiol group of the cysteine residues of OxyR may form other stable adducts *in vivo *including S-NO and S-SG, through S-nitrosylation and S-glutathionylation respectively [8]. In contrast, Lee *et al*. [9] detected no such adducts and their experiments indicated that activation of OxyR via formation of an intramolecular disulphide follows a two-step mechanism. Initially, C199 is oxidised to a sulphenic acid that is relatively unstable and rapidly reacts with C208 to form an intramolecular bond [9].

Biochemical analyses have indicated that *E. coli *OxyR is tetrameric fitting the archetype for LysR regulators [2,10] and is believed to interact with DNA as a dimer of dimers whose spacing/orientation are dependent upon the oxidation state of the protein [2]. Modelling of reduced and oxidised OxyR dimers indicates that the spacing of DNA binding domains in each dimer is unlikely to be altered by oxidation. This suggests that oxidation modulates DNA binding by modulating the dimer-dimer interaction [11].

In *N. meningitidis*, OxyR appears to both repress basal catalase activity and control enzyme induction in response to hydrogen peroxide. Thus, OxyR knock-out cells showed higher basal catalase levels than wild type cells and appeared less sensitive to hydrogen peroxide [12]. The single C199S *N. meningitidis *OxyR mutant strain behaved like the null mutant in both killing assays and primer extension analysis, in that it had neither the ability to repress or activate transcription of catalase. However, the C208A OxyR mutant strain showed an 'intermediate' phenotype; in killing assays, it was similar to wild type cells, suggesting that catalase production was repressed but in contrast to wild type cells, it was impaired in its response to hydrogen peroxide [12]. The physiological relevance of this is unclear, though it may indicate a functional role for a partially reduced form of the protein as proposed by Ieva *et al *[12].

As part of an investigation of the relationship between OxyR structure and its function in *N. meningitidis*, we have determined the crystal structure of the regulatory domain of the protein, in its authentically reduced form. The overall fold of the reduced neisserial OxyR shows a high degree of similarity to the structure of the C199S mutant OxyR (PDB code 1I69) from *E. coli*, which cannot form the redox sensitive disulphide.

By comparing the structure with those of the regulatory domains in three full length LysR structures, we have obtained some insight into how oxidation of the redox responsive disulphide may affect the quaternary structure of OxyR tetramers.

## Results

### OxyR protein production and oxidation state analysis of the cysteine residues

**Figure 1:**
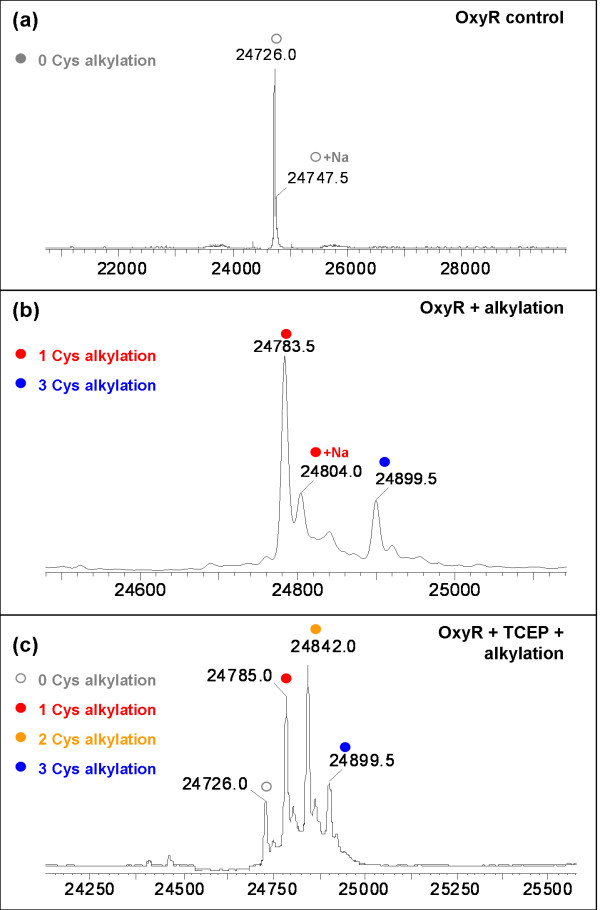
**Alkylation and MS analysis of the oxidation state of the cysteine residues of the neisserial OxyR**. Spectra: (a) OxyR control; (b) OxyR after alkylation with iodoacetamide; (c) OxyR after alkylation with iodoacetamide following prior reduction with TCEP. Minor peaks are sodium adducts of the corresponding, labelled major peaks. OxyR protein at 1 mg/ml in 20 mM Tris pH 7.5, 200 mM NaCl was incubated with/without the addition of TCEP to 2 mM at 37°C for 30 min. An equal volume of alkylating buffer (20 mM Tris pH 7.5, 0.1 mM EDTA, 20 mM iodoacetamide) was added and the samples were incubated for a further 1 h. The samples were analysed using electrospray mass spectrometry using a Dionex Ultimate liquid chromatography system connected to a Waters Q-Tof Micro mass spectrometer [24].

Selenomethionine labelled protein, corresponding to the regulatory domain of OxyR (RD-OxyR), was produced in *E. coli *for crystallization (Table 1). Crystal screening experiments were set up in both the presence and absence of the reducing agent Tris (2-carboxyethyl) phosphine (TCEP) (2 mM). Crystals only grew from the protein containing TCEP. Following this observation, we compared the oxidation state of the cysteine residues (C199, C208, and C305) in OxyR either treated with TCEP or untreated. The mass of the two protein samples was measured before and after addition of the alkylating agent, iodoacetamide (Figure 1). Prior to alkylation, the mass spectra of the untreated RD-OxyR shown in panel (a) contained two predominant peaks, with the main peak (m/z 24726) fitting with the predicted mass (24722 Da) for selenomethionine labelled protein. The small, additional peak observed at m/z 24747.5 (+21.5 Da) corresponds to a sodium adduct of RD-OxyR. No peaks consistent with any other cysteine adducts were observed. After alkylation (panel b), the main peak occurred at m/z 24783, an increase of 57 Da compared to the control sample consistent with the addition of one acetamide group (CH_2_-CO-NH_2_) to one of the cysteine residues. A secondary peak was observed at m/z 24899, corresponding to the alkylation of all three cysteine residues. The alkylation of cysteine residues is dependent on the oxidation state, and accessibility, of the reactive thiol group. When the RD-OxyR was pre-treated with TCEP prior to alkylation two additional peaks were observed (panel c). The peak at m/z 24842 corresponded to alkylation of 2 cysteine residues and the peak at m/z 24726 corresponded to unmodified RD-OxyR. The presence of these two peaks suggests that the alkylation reaction had not gone to completion in this sample.

**Table 1:** X-ray data collection and refinement statistics of OxyR (NMB0173)

Data collection details:		
X-ray source	ESRF BM14	
Data set	Peak	remote
Wavelength (Å)	0.9785	0.9070
Space group	*P2_1_*	
Unit cell (Å)	a = 49.81, b = 56.08, c = 81.25, β = 104.9°	
Resolution range (Å)	30.0 - 2.40 (2.49 - 2.40)	
Unique reflections	16661(1280)	16617(1122)
Completeness^a ^(%)	96.8(76.1)	95.1(65.4)
Redundancy	6.9(4.6)	3.9(2.8)
Average *I(/σI)*	18.4(2.6)	12.7(2.0)
R_merge_	0.129(0.554)	0.113(0.547)
Refinement statistics:		
Resolution range (Å)	30.0 - 2.40 (2.49 - 2.40)	
No. of reflections (working/test)	32077/1617	
R-factor^b^(R_work_/R_free_)	0.218/0.294	
No. of atoms (protein/water)	3254/98	
Rms bond length deviation (Å)	0.009	
Rms bond angle deviation (°)	1.3	
Mean B-factor (protein/water[Å^2^])	55.7/54.3	
Ramachandran plot:		
Most favoured regions (%)	85.5	
Allowed regions (%)	15.5	
Disallowed regions(%)	0	

^a ^The data are essentially complete to 2.6 Å resolution.

^b ^R_work _and R_free _are defined by R = Σ_hkl_||*F_obs_*|-|*F_calc_*||/Σ_hkl_|*F_obs_*|, where *h,k,l *are the indices of the reflections (used in refinement for R_work_; 5%, not used in refinement, for R_free_), *F_obs _*and *F_calc _*are the structure factors, deduced from measured intensities and calculated from the model, respectively.

Overall, the results showed that the RD-OxyR protein produced in *E. coli *contained a mixed population of proteins with cysteine residues in both reduced and oxidised forms. Although the specific proportions of each species cannot be determined from the mass spectra obtained, due to potential differences in the ionization properties of the different redox forms, the results indicate that the OxyR protein was predominantly in the oxidised state with only one cysteine available for alkylation in the absence of reduction. Given that C305 is located at the C-terminus of the protein on the opposite face of the protein to C199 and C208 (Figure 1), it seems most likely that this is the un-paired cysteine. It follows that C199 and C208 are disulphide-linked in a significant proportion of the recombinant RD-OxyR, supporting the results for "air oxidised" *E. coli *OxyR, reported by Lee *et al*. [9] which contradicted earlier data of Kim *et al*. [8] who found no evidence of a C199-C208 disulphide.

### Description of the OxyR structure

**Figure 2:**
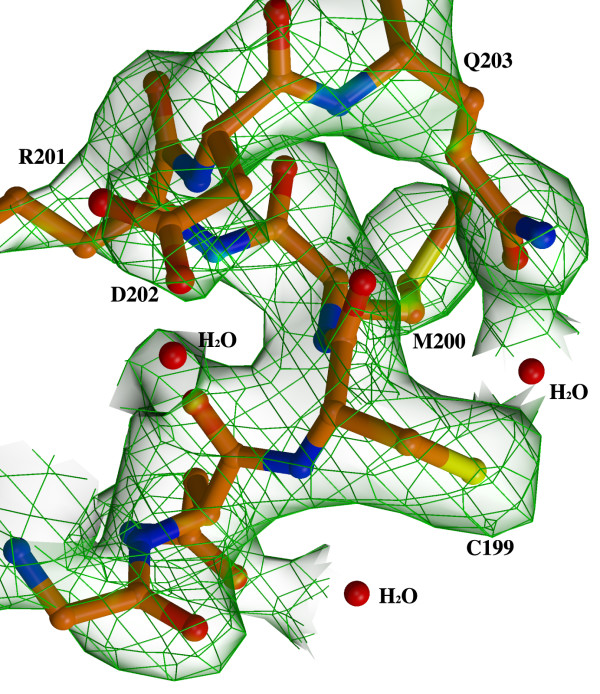
**2fo-fc map contoured at 1.0 sigma showing the electron density for part of alpha 3 helix that carrying the reduced residue Cys199**.

**Figure 3:**
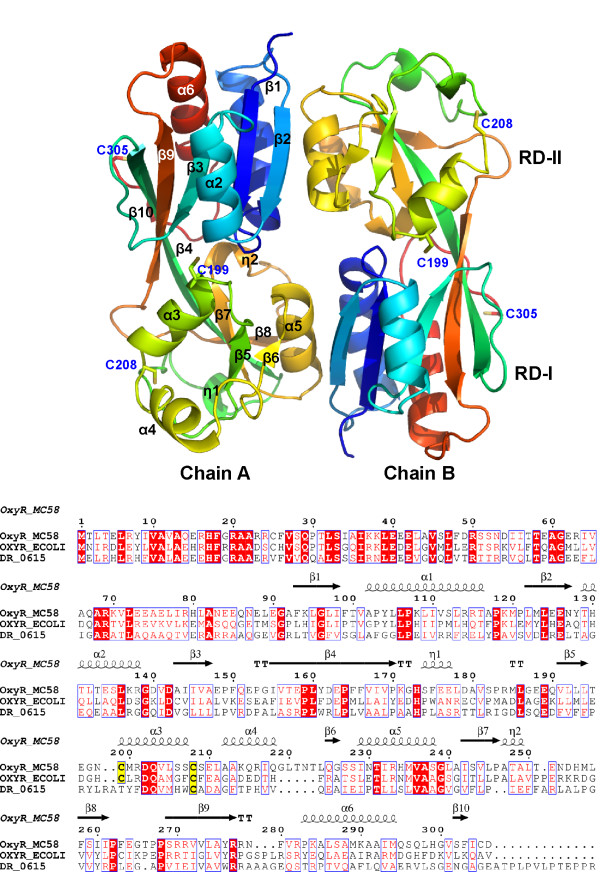
**Neisserial OxyR dimer solved to 2.4 Å resolution and multiple sequence alignment of OxyR homologues**. Abbreviations: OXYR_ECOLI, OxyR of *E. coli*; OXYR_MC58, OxyR of *N. meningitidis *MC58; DR_0615, putative OxyR (gene dr0615) from *D. radiodurans*. The secondary structural elements of the neisserial OxyR are shown above the alignment.

The *N. meningitidis *RD-OxyR structure was determined to a resolution of 2.4 Å by multiple wavelength anomalous dispersion method using a selenomethionine-substituted protein. As expected from the requirement for TCEP during crystallization, the protein was in the reduced form with no disulphide between C199 and C208. We observed no evidence for partial reduction of C199 in the electron density (Figure 2). The asymmetric unit contained a dimer arranged in the classical head-to-tail orientation, typical for regulatory domain structures of LysR transcription factors (Figure 3) which was first observed in the structure of the regulatory domain of *E. coli *CysB [13].

**Figure 4:**
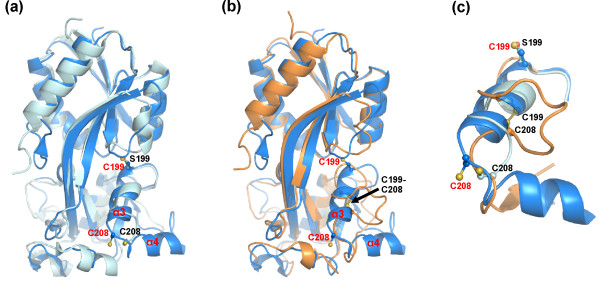
**Structural comparison of the *N. meningitidis *OxyR and *E. coli *OxyR**. (a) Superimposition of the *N. meningitidis *OxyR (blue) and the 'reduced' C199S *E. coli *OxyR (grey, PDB code 1I69). (b) Superimposition of the *N. meningitidis *OxyR (blue) and the oxidised *E. coli *OxyR (orange, PDB code 1I6A). (c) Comparison of the redox active centre of, *N. meningitidis *OxyR (blue); 'reduced' C199S *E. coli *OxyR (grey); and oxidised *E. coli *OxyR (orange).

Each OxyR chain comprises two sub-domains, RD-I (residues 90-162 and 270-306) and RD-II (residues 163-269) which contains the redox centre (C199 and C208). There are three residues missing from both the N- and C-termini in the final model of the first monomer, (chain A residues 88-89 and 307). RD-I, which is more structurally conserved within the LysR family compared to RD-II, contains secondary structural elements of topology β1-α1-β2-α3-β3-t-β4 from the N-termini and β9-t-α6-β10 from the C-termini. The β4 and β9 strands cross-over between the two sub-domains. RD-II contains a central core of 4 β strands (β5-β8) surrounded by a series of 3^10 ^(η1-η2) and α helices (α3-α5) (Figure 3). The two redox sensitive cysteines (measured from the γS of C199 and C208, chain A) are separated by 18.4 Å and are located either end of helix α3 (C199-S207). Residues A212-Q218 form a second helix (α4) which extends out of RD-II, at an angle of ~75° relative to α3 (Figure 4a). Chain B is missing 20 residues in total (residues 88-89, 209-221 and 307) largely from RD-II. Disorder within this region of the regulatory domain was observed in both chains of the structure of the *E. coli *C199S OxyR mutant [2].

### Comparison of the *E. coli *and *N. meningitidis *OxyR regulatory domain structures

The structure of reduced *N. meningitidis *OxyR (Chain A) was compared to that of the C199S OxyR mutant from *E. coli *(PDB code 1I69, chain B) which represents a 'pseudo reduced' form of the protein. Overall, the two structures proved to be very similar, superimposing with a root mean square deviation (RMSD) of 1.57 Å for 193 equivalent Cα atoms. In the overlay shown in Figure 4a, residue S199 of the 'pseudo reduced' *E. coli *structure and the corresponding C199 residue of neisserial OxyR are in almost exactly the same position. The side-chains of both residues are directed towards the interior of the protein, though C199 is more exposed to solvent, with a calculated accessible surface area (ASA) of 32 Å^2 ^compared to 14 Å^2 ^for S199. Choi *et al *propose that in the reduced state, C199 in *E. coli *OxyR is stabilised by hydrophobic interactions involving L200, L224 and L224 and proximity to R266 which could form a charge interaction with an ionised C199 [2]. The corresponding residues (M200, I229, P246 and R270) are structurally conserved within neisserial OxyR, indicating that they are likely to have the same stabilising affect in this protein. By contrast, the side-chains of C208 are pointing in different directions in the two structures, with C208 buried in neisserial OxyR with an ASA of 5 Å^2 ^compared to 109 Å^2 ^for the surface exposed C208 in *E. coli *OxyR. Reactivity of C199 with peroxide is believed to initiate the redox switch in OxyR with oxidation to a sulphenic acid [9]. Formation of the disulphide with C208 requires significant local rearrangement of the polypeptide chain. In the structure of oxidised *E. coli *OxyR (PDB code 1I6A), the helix equivalent to the α3 helix in the neisserial structure (Figures 3 and 4) is unwound adopting a random coil type arrangement. Presumably, a similar conformational change would occur on oxidation of the *N. meningitidis *OxyR. Interestingly, residues that follow on from α3 helix in the reduced *N. meningitidis *OxyR structure (A212-Q218 in Chain A) form an alpha helix (α4), whereas the equivalent residues in the *E. coli *C199S structure are disordered (PDB code 1I69, chain A, residues 210-215 and chain B, residues 211-216, are absent from the final model). In the *N. meningitidis *OxyR sequence there is an insertion following the α4 helix which is absent from the *E. coli *sequence and may account for the structural differences in this region (Figure 3). In the structure of oxidised *E. coli *OxyR, residues corresponding to helix α4 in the neisserial structure adopt a different secondary structure namely a short beta strand (residues, D214-D216) (Figure 4c). Whether helix α4 in neisserial OxyR would be preserved following formation of the C199-C208 disulphide is uncertain, though examination of the reduced *N. meningitidis *OxyR structure suggests that helix α4 could be re-positioned without loss of secondary structure.

### Modelling the full length OxyR

Biophysical characterization of full-length *E. coli *OxyR indicates that it is tetrameric in solution [10,14]. Furthermore, a C199S mutant that models the reduced state of the protein also behaves predominantly as a tetramer according to size exclusion chromatography [10]. Thus, OxyR appears to be typical of LysR family regulators which are generally observed to form tetramers (e.g. Dntr [15], OccR [16] and CysB [17]). An exception to this is the CrgA regulator, also from *N. meningitidis*, which we have previously shown to be octameric in solution and in the crystal structure [18]. Cluster analysis of all known LysR sequences has shown that CrgA belongs to a sub-group that is distinct from the majority of LysR including OxyR [18].

**Figure 5:**
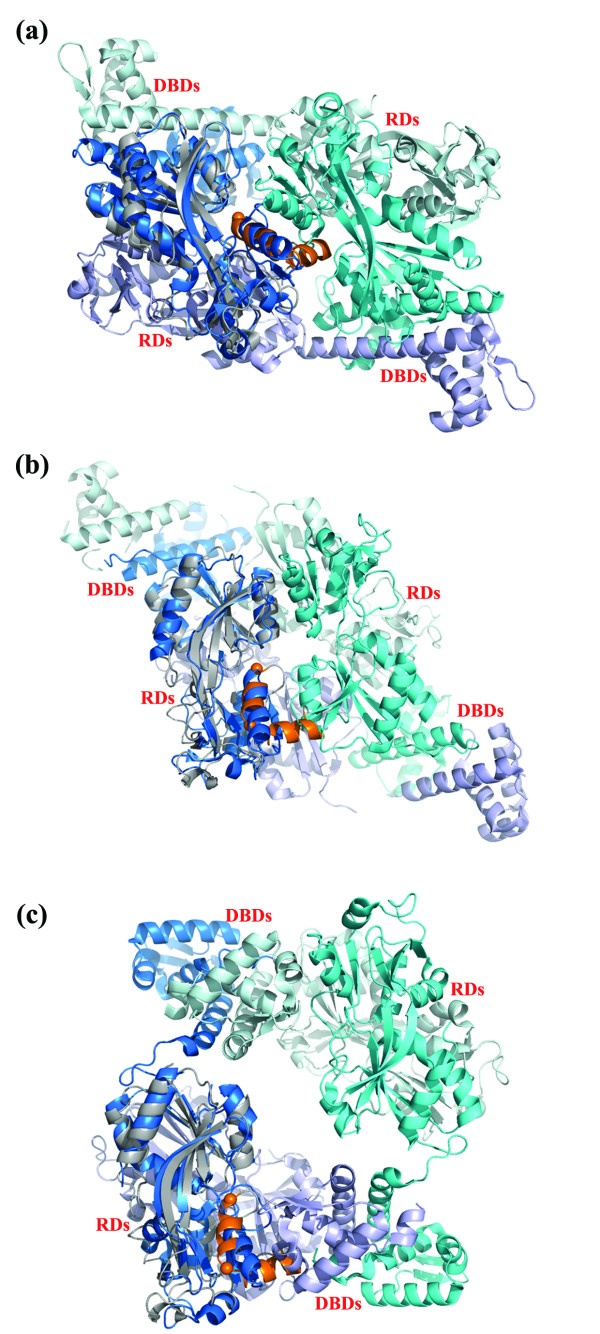
**Modelling of a neisserial OxyR tetramer**. The OxyR regulatory domain shown in grey was superimposed onto the tetrameric structures of (a) CbnR (PDB code 1IZ1). (b) PA01 (PDB id 3FZV) and (c) PA0477 (PDB id 2ESN). The α3 and α4 helices which flank the C199 and C208 residues (shown as solid spheres) are coloured orange. The two pairs of dimers which form each tetramer are coloured bright/light blue (A:C in PA01 and PA0477; A:Q in CbnR) and bright/light cyan (B:D in PA01 and PA0477; B:P in CbnR).

To visualise how the reduced form of neisserial RD-OxyR might be arranged in the full length protein, we superimposed the structure onto that of three LysR tetramers whose crystal structures have been determined. These were CbnR from *Ralstonia eutropha *NH9 which regulates catechol metabolism ([19] PDB id 1IZ1) and two putative LysR transcription factors from *Pseudomonas aeruginosa *(PA0477, PDB id 2ESN and PA01, PDB id 3FZV unpublished data). Although each tetramer consists of a dimer of dimers, the arrangement of the dimers in each tetrameric assembly is significantly different. This in turn determines the relative positions of the N-terminal DNA binding domains. The OxyR regulatory domain superimposed well on each structure, with a RMSD of ~2.0 Å for 160 (2ESN), 171 (1IZ1) and 175 (3FZV) equivalent Cα atoms (Figure 5) and corresponding sequence identities of 15.6% (2ESN), 17.5% (1IZ1) and 24.6% (3FZV) respectively From a visual inspection of the overlayed structures, it is possible to see how formation of the C199-208 on oxidation of OxyR could affect the quaternary arrangement of the protein. As described below, the situation is different for each form of tetrameric assembly represented by the three structures. In the CbnR structure, each dimer, formed between chains A & Q, or B & P, contains one monomer in a compact form (chains A and P) and one in an extended conformation (chains B and Q). The only contact between the regulatory domains of the two dimers is found in the core of the tetramer, at the interface formed primarily between the C-terminal α helices of the two RD-II sub-domains of chains A & P (Figure 5a). The overlay with reduced OxyR shows that the helices, α3 and α4 which are located either side of C208 would contribute to the dimer-dimer interface in this model of OxyR (Figure 5a). Therefore, the formation of the C199-C208 disulphide bridge which involves a disruption of the α3 helix (Figure 4 and [2]) would be expected to alter the dimer-dimer interface and hence the quaternary structure of the OxyR tetramer. In the PA01 tetramer, the regulatory domains also contribute to the dimer-dimer interface, but in this case the domains of all four subunits are involved (Figure 5b). In a "head-to-tail" arrangement of the regulatory domains of the two dimers, the α3 and α4 helices of the overlayed reduced OxyR still form part of the dimer-dimer interface (A:C and B:D, Figure 5b) packing onto residues H153-I159 (residues in PA01) in RDI of the adjacent dimer. Again, formation of the disulphide would be anticipated to alter the dimer-dimer packing. The PA0477 presents a very different tetrameric arrangement compared to the other two LysR structures in that there is no contact between the regulatory domains of the two dimers (A:C and B:D Figure 5c) rather the dimer-dimer interfaces are formed by the DNA binding domains and interdomain linker helices. In this case, superimposition of the reduced OxyR regulatory domain indicates that the α3 and α4 helices contribute to the interface between adjacent subunit monomers packing onto the second helix of the DNA binding domain (A and C, B and D Figure 5c).

In the absence of a full length OxyR structure we do not know which (if any) of the three structures we have considered provides the best model of the OxyR tetramer. It may well be that OxyR can adopt all the configurations represented by the three structures. However in two of the three possible models (Figure 5a and 5b) the redox sensitive disulphide is located at a dimer-dimer interface. This provides a plausible explanation of how formation of the disulphide in response to oxidative challenge, may modulate the arrangement of subunits in the tetrameric assembly. This in turn would affect the relative positions of the DNA binding domains and hence account for the changes in the interaction with target promoters that has been observed experimentally. The oxidation of C199-C208 would have a more direct effect on the position or orientation of the DNA binding domains if OxyR adopted the tetrameric configuration of PA0477 (Figure 5c).

## Discussion

As reviewed by Paget and Buttner in 2003 [20] two different mechanisms for OxyR regulation have been proposed by the Stamler and Storz research groups respectively. The Storz group suggest that OxyR functions as an on-off switch, converting between oxidised, with an intramolecular disulphide, and reduced forms; whereas the Stamler group, who detected no disulphide bond, suggest that OxyR has a graded response with C199 being subject to different modifications (e.g. S-OH, SNO or S-SG) producing different transcriptional outcomes [20]. Consistent with the results of Storz *et al*., our mass spectrometry data indicated that the nessierial OxyR contains a disulphide presumably the result of air oxidation during purification; no cysteine adducts were detected. This favours an activation mechanism based on the reversible oxidation of the conserved cysteine pair (residues 199 and 208). Molecular modelling with the reduced and oxidised OxyR structures indicated that formation of such an intramolecular disulphide bond would affect the dimer-dimer interface of the neisserial OxyR tetramer. A small movement at this pivot point, in the centre of the tetramer, could potentially translate into a much larger movement of the heads of the DNA binding domains (Figure 5). An indication that oxidation of OxyR is associated with a change in DNA binding comes from studies of *E. coli *OxyR. Wild type and C199S OxyR mutant were compared in gel shift and foot printing experiments and shown to differ in their DNA binding properties [11]. In the case of its own promoter, the results showed that the oxidised form of the protein contacted the *oxyR *promoter at four consecutive major grooves consistent with a tetrameric assembly. By contrast the C199S mutant protected a more extended region containing two binding sites, each comprising two major grooves separated by one helical turn. This indicates that the oxidised form of *E. coli *OxyR adopts a more compact structure, with the DNA binding pairs closer together, compared to the reduced form [14]. Although the *N. meningiditis *OxyR structure provides some clues as to how oxidation of OxyR could alter its interaction with DNA, it does not readily explain the phenotypic differences between the single C199S and C208A mutant neisserial strains reported by Ieva *et al*. [12]. The C199S mutant does not appear to be functionally active (i.e. it cannot repress or activate expression) whereas the C208A is able to repress but not activate catalase expression. Formation of the C199-C208 disulphide bond is presumably required for activation of the *kat *promoter since this is blocked by mutation of either cysteine residue. However, repression of the *kat *promoter only requires C199 since the C208A mutant retains repressive activity whereas the C199S is inactive. To ascertain whether this reflects conformational differences between the two proteins will require further structural studies and comparison with the authentically reduced protein reported here. Intriguingly, an OxyR homologue from *Deinococcus radiodurans *has recently been reported that contains only one of the conserved cysteines (C210), corresponding to the C208 of *E. coli *and *N. meningitidis *OxyR (Figure 2) [21]. *Deinococcus *strains in which the *oxyR *gene had been either deleted or substituted with a C210A mutant gene, showed increased sensitivity to hydrogen peroxide challenge and no increase in catalase activity compared to wild type bacteria. C210 residue was also shown to be oxidised to a sulphenic acid *in vitro *under conditions of oxidative stress [21]. It would be very interesting to compare the structure of the *Deinococcus *OxyR with those of *N. meningitidis *and *E. coli *in order to understand better the role of the individual cysteines in the functional responses of these proteins.

## Conclusions

The structure of the regulatory domain of *N. meningitidis *OxyR represents the first determined for a wildtype OxyR protein in its reduced form. Although there are subtle differences, overall, the structures of neisserial and *E. coli *OxyR are very similar and hence the mechanism of activation of these regulators is likely to be similar in both species. Modelling of an *N. meningitidis *OxyR tetramer suggested that the redox active cysteines, C199 and C208, are located at the interface of the two dimers that form the LysR tetramer. This location suggests that formation of the C199-C208 disulphide would affect the relative disposition of the two dimers within the tetramer which in turn may alter the spacing/orientation of the DNA binding domains and hence the interaction of OxyR with its DNA binding sites.

## Methods

### Protein production

The *oxyR *gene (NMB0173) was amplified from genomic DNA using the forward primer 5'-AAGTTCTGTTTCAGGGCCCGGAGCTGGAGGGTGCGTTCAAAC-3' and reverse primer 5'-ATGGTCTAGAAAGCTTTACTAGTCGCAGATAAAAC TTACCCCG-3' and cloned into pOPINF using In-Fusion™ cloning technology as reported [22]. Selenomethionine labelled protein was produced from the pOPINF OxyR expression construct containing OxyR residues 88-307 and an N-terminal cleavable His tag [MAHHHHHHSSGLEVLFQ↓GP, (↓ human rhinovirus 3C protease cleavage site)] in *E. coli *strain B834 using SelenoMet media (Molecular dimensions). The recombinant protein was purified using Nickel affinity chromatography and subsequently size exclusion chromatography following methods previously described [23]. 100% selenium incorporation was confirmed by mass spectrometry [24].

### Crystallography and structure solution

After removal of the N-terminal His tag, the selenomethionine labelled OxyR protein was concentrated to 9 mg/ml in 20 mM Tris pH 7.5, 200 mM NaCl. Fractions were pooled and concentrated in Vivaspin 15 concentrators (Vivascience). Crystallization screening experiments in nanodrops were performed as described [25]. Following unsuccessful initial crystallization screening experiments, further crystal trials were set up with the same batch of protein, but with 2 mM of the reducing agent, TCEP, present. Under these conditions the protein crystallized and the crystals were subsequently optimized using the standard three row optimization procedure detailed by Walter *et al*. [26]. The crystal used for data collection grew in a drop of 200 nl protein and 100 nl of reservoir solution containing 20% w/v PEG 3350, 0.2 M calcium acetate (Hampton PEG/Ion Screen, condition 28). The crystal was flash frozen in a cryoprotectant solution of 25% v/v ethylene glycol and 75% of the reservoir solution and maintained at 100 K under a stream of nitrogen gas during data collection. Multiple wavelength anomalous dispersion data were collected at two wavelengths at beamline BM14, the ESRF (Grenoble, France) to 2.4 Å resolution. The HKL2000 suite was used to integrate and scale the data [27]. The SHELX program suite was used to monitor the anomalous signal and to identify the selenium sites [28]. SOLVE/RESOLVE [29] were then used for refinement of selenium positions and phase improvement, combined with automated model building. Refinement was carried out with CNS [30] using simulated annealing and positional refinement with main chain NCS restraints, followed by individual isotropic B factor refinement. The final stages of refinement were carried out with PHENIX using anomalous scattering factors [31]. The coordinates and structure factors have been deposited in the Protein Data Bank under accession number 3JV9.

### Bioinformatics and structure analysis

Structural alignments were carried out using the programme SHP [32]. The figures were produced using Pymol [33]. Sequences were aligned using ClustalW [34] and displayed with secondary structures using ESPript2.2 [35].

### MS analysis of the oxidation state of cysteine residues

OxyR protein at 1 mg/ml in 20 mM Tris pH 7.5, 200 mM NaCl was incubated with/without the addition of TCEP to 2 mM at 37°C for 30 min. An equal volume of alkylating buffer (20 mM Tris pH 7.5, 0.1 mM EDTA, 20 mM iodoacetamide) was added and the samples were incubated for a further 1 h. The samples were analysed using electrospray mass spectrometry using a Dionex Ultimate liquid chromatography system connected to a Waters Q-Tof Micro mass spectrometer [24].

## Authors' contributions

NJS, DIS and RJO initiated the study. SS cloned, purified and crystallized the protein, JN carried out analysis by mass spectrometry, JR and SS collected and processed the diffraction data, modelled and refined the structure. SS, JR and RJO analysed the structure and wrote the paper which was read and approved by all authors.
